# Drug discovery in advanced and recurrent endometrial cancer: Recent advances

**DOI:** 10.32604/or.2025.061120

**Published:** 2025-06-26

**Authors:** ALEX A. FRANCOEUR, NATALIE AYOUB, DANIELLE GREENBERG, KRISHNANSU S. TEWARI

**Affiliations:** Department of Obstetrics and Gynecology, University of California, Irvine, Orange, CA 92868, USA

**Keywords:** Advanced endometrial cancer, Recurrent endometrial cancer, Drug discovery, Tumor cancer genome atlas, Targeted therapy, Chemotherapy, immunotherapy

## Abstract

Endometrial cancer is the most common gynecologic cancer diagnosed in the United States and mortality is on the rise. Advanced and recurrent endometrial cancer represents a treatment challenge as historically there have been limited therapeutic options for patients. In the last several years, multiple practice-changing clinical trials have led to significant improvements in the treatment landscape. This review will cover updates in the treatment and management of advanced and recurrent endometrial cancer with a focus on novel therapeutics, such as anti-PD-L1 and PD-1 inhibitors, poly ADP-ribose polymerase (PARP) inhibitors, antibody-drug conjugates, and hormonal therapy.

## Introduction

Endometrial cancer (EC) is the most common gynecologic malignancy diagnosed in the United States with 67,000 cases estimated to be diagnosed in 2024 [1,2]. Further, it carries a growing degree of morbidity and mortality associated with it; EC was projected to surpass ovarian cancer as the most deadly gynecologic malignancy in 2024 [1]. A large degree of mortality is driven by increasing rates of high-risk histologic subtypes of EC such as serous carcinoma, clear cell carcinoma, and carcinosarcoma. These cancers are typically diagnosed at a more advanced stage and carry a poor prognosis [3,4]. For example, 23% of serous uterine cancers and 17% of clear cell carcinomas are diagnosed at an advanced stage compared to 4% of endometrioid cancers [5]. Rates of carcinosarcomas are increasing in the United States, rising 3-fold over the last four decades [6]. Further, the incidence of these histologic subtypes disproportionately affects minority populations within the United States. Black women have a 3–4-fold higher rate of serous carcinoma and carcinosarcoma compared to White women [7,8] In this review, changes in the management of locally advanced, metastatic, and recurrent endometrial cancer will be covered with a focus on phase 3 trials reported on or published in the last several years. Additionally, this review will examine how the molecular understanding of EC has shaped treatment and research paradigms. Finally, some relevant phases 1 and 2 clinical trials and advances made in radiotherapy will be examined.

## Methods

All relevant phase 3 trials presented on or published related to locally advanced, metastatic, or recurrent endometrial cancer from 2020 to the present were included in this review. For phases 1 and 2 trials, a literature search of clinicaltrials.gov and PubMed was performed (September–October 2024) with the decision to include phase 1 or 2 trials based on expert opinion.

### Historical treatment of advanced and recurrent endometrial cancer

Chemotherapy was identified to be active in advanced and recurrent EC as far back as the 1970s. Initially, cisplatin and doxorubicin were identified to elicit a good response in the treatment of these patients [9,10]. This led to GOG-107, where patients with stage III or IV primary or recurrent EC were randomized to treatment with doxorubicin 60 mg/m^2^ every 3 weeks or doxorubicin and cisplatin 50 mg/m^2^ until disease progression or a maximum of 500 mg/m^2^ of doxorubicin [11]. They reported increased activity with an overall response rate (ORR) of 42% in the doxorubicin and cisplatin arm compared to 25% with doxorubicin alone (*p* = 0.004). Progression-free survival (PFS) was improved with combination therapy with a median PFS (mPFS) of 5.7 vs. 3.8 months (Hazard ratio (HR) 0.74, 95% CI: 0.58–0.94). However, there was no difference in overall survival (OS) among the two arms; 9.0 months compared to 9.2 months. This trial demonstrated the promise of combining chemotherapeutic agents for an enhanced effect. In GOG-163, researchers compared the previous cisplatin-doxorubicin doublet to doxorubicin and paclitaxel 150 mg/m^2^ over 24 h with filgrastim in a similar patient population [12]. No difference in ORR, PFS, or OS was found with the substitution of cisplatin for paclitaxel.

From here researchers examined if triple therapy could provide a further synergistic benefit in GOG-177, where cisplatin, doxorubicin, and paclitaxel (TAP) were compared to cisplatin and doxorubicin alone [13]. A similar patient cohort as in GOG-107 and GOG-163 was enrolled. The addition of paclitaxel to cisplatin and doxorubicin resulted in a statistically significant improvement in ORR (57% vs. 34%, *p* < 0.01), PFS (8.3 vs. 5.3 months, *p* < 0.01), and OS (15.3 vs. 12.3 months, *p* < 0.03). The TAP regimen was not without drawbacks; it had a high level of toxicity, especially neuropathy. This led investigators to compare TAP to carboplatin and paclitaxel, a regimen well-established and tolerated in ovarian cancer [14]. GOG-0209 (NCT00063999) examined the non-inferiority of carboplatin and paclitaxel (TC) to TAP [15]. They enrolled over 1300 patients with stage 3, 4, or recurrent EC and randomized them to carboplatin area under the curve (AUC) 6 and paclitaxel 175 mg/m^2^ or the previously described TAP regimen. The median OS of TC was 37 months compared to 41 months in TAP and was determined to be non-inferior (HR: 1.002, 90% CI: 0.90–1.12). Median PFS was not statistically significantly different. Toxicities and quality of life outcomes favored TC, and since 2012, carboplatin and paclitaxel has been the backbone of treatment for advanced and recurrent EC. With a median overall survival of less than two years, patients with this disease still face a poor prognosis and have limited subsequent treatment options after progression on carboplatin and paclitaxel.

*Summary:* GOG-0209 established carboplatin and paclitaxel as the backbone of treatment for advanced or recurrent EC but long-term outcomes remained poor.

### Advancements in the molecular understanding and staging of endometrial cancer

#### The molecular classification of endometrial cancer

In 2013, Levine et al., published the practice changing Cancer Genome Atlas study in which the molecular classification of EC was introduced [16]. In the Levine study, four subgroups of endometrial tumors were identified based on integrated genomics data: DNA polymerase epsilon catalytic subunit (POLE) hypermutated, microsatellite instability-high/mismatch repair deficient (MSI high/dMMR), copy number low/TP53 wildtype/no specific molecular profile (CNL/TP53 WT/NSMP), and copy number high/TP53 abnormal (CNH/TP53 ABN). These four subgroups were characterized by specific patterns in the genomics, transcriptomics, and proteomics of EC that have been associated with prognosis and treatment response in several retrospective studies [17–19].

The genomic analysis performed in the TCGA requires fresh specimens and a complex and costly methodology that does not have real-world applicability [16]. This led researchers to develop the pragmatic molecular classification tool (ProMisE), where formalin-fixed paraffin-embedded specimens and more readily available staining techniques can be utilized [20]. The algorithm uses immunohistochemistry (IHC) to evaluate for the presence or absence of mismatch repair proteins for MMR deficiency, sequencing for POLE exonuclease domain mutations, and TP53 IHC to classify TP53 ABN and TP53 WT [21]. This algorithm has been validated and allows for more real-world applicability. Molecular testing can be time-consuming and costly, especially in low-resource settings. Studies have found that the inclusion of POLE testing with treatment de-escalation can reduce cost while ProMisE algorithm testing can increase cost with treatment escalation for TP53 ABN tumors [22,23]. DNA sequencing for POLE status can be costly and the development of rapid, cost-effective options such as qPCR is essential for widespread use and dissemination [24].

PORTEC-3 was one of the first clinical trials to retrospectively analyze outcomes based on molecular subgroups [25,26]. In PORTEC-3, the role of combined adjuvant chemoradiation (CRT) was compared to adjuvant radiation (RT) alone in a high-risk EC cohort. A significant difference in treatment response was observed when patients were retrospectively stratified into TCGA subgroups based on the ProMisE algorithm. Patients with TP53 abn tumors showed a benefit in relapse-free survival (RFS) with CRT compared to RT alone (59% vs. 36%, *p* = 0.019), while patients with POLE hypermutated tumors had an excellent prognosis regardless of treatment (RFS 100% vs. 97%, *p* = 0.637), and patients with dMMR tumors demonstrated no difference in RFS (68% vs. 76%, *p* = 0.428). These results suggest that an escalation of adjuvant treatment may be indicated in patients with TP53 abn tumors while de-escalation of treatment may be permissible in patients with POLE hypermutated tumors.

A recent ancillary analysis of GOG-258 presented in 2024 and published in 2025 also demonstrated differences in outcomes by molecular subgroup based on a modified ProMisE algorithm [27]. In the original GOG-258 phase 3 trial, patients with advanced-stage EC were randomized to receive CRT (similar to PORTEC-3) vs. chemotherapy alone (six cycles of carboplatin/paclitaxel). No significant difference was seen in the primary outcome, RFS or OS between the two cohorts [28]. The post-hoc analysis stratified patients into p53 WT, p53 abn, and dMMR molecular subgroups. The p53 WT cohort who received CRT showed an improved RFS and OS in comparison to chemotherapy alone (77.2% vs. 59.7%; HR = 0.54, 95% CI: 0.32–0.94; *p* = 0.02). No difference in RFS or OS was observed within the p53 abn (29.3% vs. 29.4%; 95% CI: 0.46–1.24) or dMMR subgroups (52.5% vs. 63.7%, 95% CI: 0.70–2.57) [27]. These results demonstrate a need for further prospective studies, such as the ongoing PORTEC-4a (NCT03469674) and RAINBO studies (NCT05255653), to evaluate therapies based on molecular classification [29] (Table 1).

**Table 1:** Summary of relevant ongoing clinical trials in the treatment of advanced and recurrent endometrial cancer

Trial name	Objective	Study design	Status
PORTEC-4aNCT03469674	To compare rates of vaginal recurrence in patients with high-intermediate risk endometrial cancer, assessing outcomes based on recommendations from a molecular-integrated risk profile for adjuvant therapy	Phase 3	Ongoing
RAINBO NCT05255653	To compare treatment responses of endometrial cancer based on the patient’s tumor genome atlas classification	Phase 2/3	Ongoing
	p53abn-RED trial (NCT05255653-1): adjuvant CRT followed by olaparib for two years is compared to adjuvant CRT		
	MMRd-GREEN trial (NCT05255653-2): adjuvant pelvic EBRT combined with and followed by durvalumab for one year is compared to adjuvant pelvic EBRT		
	NSMP-ORANGE trial (NCT05255653-3): adjuvant pelvic EBRT followed by progestogens for two years is compared to adjuvant CRT		
	POLEmut-BLUE trial (NCT05255653-4): safety of de-escalation of adjuvant therapy is investigated: no adjuvant therapy for stage I-II disease and no adjuvant therapy or pelvic EBRT only for stage III disease		
TroFuse-005 MK-2870-005/ENGOT-en23/GOG-3095NCT06132958	To compare MK-2870 sacituzumab irinotecan to chemotherapy in patients with recurrent and advanced endometrial cancer with prior chemotherapy and immunotherapy	Phase 3	Ongoing
ASCENT-GYN-01/GOG-3104/ENGOT-en26 NCT06486441	To compare sacituzumab govitecan to chemotherapy in patients with recurrent and advanced endometrial cancer with prior chemotherapy and immunotherapy	Phase 3	Ongoing
XPORT-EC-042/GOG-3083/ENGOT-EN20 NCT05611931	To compare selinexor maintenance to placebo in patients with TP53 wild-type advanced or recurrent endometrial cancer after treatment with platinum-based chemotherapy	Phase 3	Ongoing
KRT-232-118/ENGOT en-21/GOG-3089 NCT05797831	To compare navtemadlin maintenance to placebo in patients with TP53 wild-type advanced or recurrent endometrial cancer after treatment with platinum-based chemotherapy	Phase 2/3	Ongoing
NRG-GY026NCT05256225	To compare trastuzumab/hyaluronidase-oysk and paclitaxel with trastuzumab/hyaluronidase-oysk maintenance to carboplatin, paclitaxel, and pertuzumab/trastuzumab/hyaluronidase-zzxf and pertuzumab/trastuzumab/hyaluronidase-zzxf maintenance to carboplatin and paclitaxel in patients with Human epidermal growth factor 2 (HER-2) positive serous carcinoma or carcinosarcoma	Phase 2/3	Ongoing
Futibatinib and pembrolizumab NCT05036681	To evaluate the objective response rate (ORR) of futibatinib and pembrolizumab in patients with metastatic microsatellite stable (MSS) endometrial carcinoma	Phase 2	Ongoing
ALPINE NCT06366347	To evaluate the use of maintenance letrozole + abemaciclib vs. pembrolizumab after carboplatin + paclitaxel + pembrolizumab chemotherapy in participants with advanced or recurrent estrogen receptor-positive, mismatch repair proficient, TP53 wild-type endometrial cancer	Phase 2	Ongoing
TSR-042 in addition to standard of care definitive radiation for inoperable EC NCT03955978	To evaluate the use of anti-PDL1 therapy in combination with standard radiation therapy in newly diagnosed, medically inoperable endometrial cancer	Phase 1	Ongoing
Lenvatinib, pembrolizumab, and hypofractionated pelvic RT NCT05603910	To evaluate the use of lenvatinib in combination with pemrolizumab and hypofractionated pelvic radiation therapy in patients with recurrent or unresectable pMMR endometrial cancer	Phase 1	Ongoing

#### Changes to the FIGO staging of endometrial cancer

Based on the findings from the TCGA, the classification of EC has shifted from the traditional paradigm of Type 1 and Type 2 EC [17]. The International Federation of Gynecology and Obstetrics (FIGO) published a new staging system in 2023, incorporating the TCGA molecular classification system into staging [30]. In the 2023 guidelines, certain molecular subgroups may upstage or downstage a patient’s disease (Table 2).

**Table 2:** Comparison between FIGO 2009 and FIGO 2023 endometrial cancer staging

Stage	2009 FIGO staging [33]	2023 FIGO staging [30]
**I**	Confined to the corpus uteri	Confined to the uterine corpus and ovary
	IA no or <50% myometrial invasion	IA disease limited to the endometrium or non-aggressive histological type, i.e., low-grade endometrioid, with <50% myometrial invasion with no or focal lymphovascular space involvement (LVSI) or good prognosis disease
		IA1 non-aggressive histological type limited to an endometrial polyp or confined to the endometrium
		IA2 non-aggressive histological types involving less than half of the myometrium with no or focal LVSI
		IA3 low-grade endometrioid carcinomas limited to the uterus and ovary
	IB invasion equal to or more than half of the myometrium	IB non-aggressive histological types with invasion of half or more of the myometrium, and with no or focal LVSI
**II**	Tumor invades cervical stroma but does not extend beyond the uterus	Invasion of cervical stroma without extrauterine extension or with substantial LVSI or aggressive histological types with myometrial invasion
		IIA invasion of the cervical stroma of non-aggressive histological types
		IIB substantial LVSI of non-aggressive histological types
		IIC aggressive histological types with any myometrial involvement
**III**	Local and/or regional spread of the tumor of any histological subtype	
	IIIA invasion of uterine serosa, adnexa, or both by direct extension or metastasis	
	IIIB vaginal and/or parametrial involvement	IIIA1 spread to the ovary or fallopian tube (except when meeting stage IA3 criteria)
		IIIA2 involvement of uterine subserosa or spread through the uterine serosa
		IIIB1 metastasis or direct spread to the vagina and/or the parametria
		IIIB2 metastasis to the pelvic peritoneum
	IIIC metastases to pelvic and/or para-aortic lymph nodes	
	IIIC1 positive pelvic nodes	
		IIIC1i micrometastasis
		IIIC1iI macrometastasis
	IIIC2 positive para-aortic lymph nodes up to the renal vessels with or without positive pelvic lymph nodes	
		IIIC2i micrometastasis
		IIIC2iI macrometastasis
**IV**	Spread to the bladder mucosa and/or intestinal mucosa and/or distance metastasis	
	IVA tumor invasion of bladder and/or bowel mucosa	
	IVB distant metastases, including intra-abdominal metastases and/or inguinal lymph nodes	IVB abdominal peritoneal metastasis beyond the pelvis
		IVC distant metastasis, including metastasis to any extra- or intra-abdominal lymph nodes above the renal vessels, lungs, liver, brain, or bone

These guidelines have been met with significant controversy. Support for this shift emphasizes the prognostic importance of molecular classification and its utility in personalized treatment. The PORTEC-3 and GOG-258 post-hoc analyses highlight the growing importance of molecular classification in personalizing treatment for EC. Criticisms of the new staging system include inconsistent access to molecular testing in low-resource settings, lack of a standardized protocol for molecular testing, and limited prospective data for application in the clinical setting [31,32]. Prospective trials are still needed to validate these changes to the staging guidelines. However, given the overwhelming number of retrospective studies favoring molecular classification and the numerous ongoing clinical trials evaluating treatment response by molecular subtypes (NCT03469674, NCT05640999, NCT05255653, NCT04214067, NCT04159155, NCT04634877), certain aspects of the new staging guidelines are likely to become routine in clinical practice in the coming years.

*Summary:* The understanding of the molecular underpinnings of EC has helped advance research and drug development. Current, ongoing studies will help practitioners understand if treatment for EC can be personalized based on mutations the tumors carry. More work is needed to make molecular classification of EC globally accessible.

## Immunotherapy in Endometrial Cancer

Approximately one-third of patients with EC have tumors with mismatch repair deficiency or high levels of microsatellite instability (dMMR/MSI-H) [34]. Mismatch repair deficiency can occur due to somatic tumor mutations, epigenetic alterations within tumor cells, or inherited mutations, known as Lynch Syndrome [35]. The most common gene affected by sporadic methylation is the mutL homolog 1 (MLH1) promoter. Other affected genes either via sporadic or inherited mutations include postmeitotic segregation increased 2 (PMS2), mutS homolog 2 (MSH2), and mutS homolog 6 (MSH6) [35]. Mutations in the aforementioned genes prevent recognition, excision, and resynthesis of DNA errors, leading to an accumulation of point mutations within microsatellite regions, and the development of microsatellite instability [36]. MSI-H/dMMR endometrial cancers are often associated with an increased number of neoantigens, PD-1 expression on tumor-infiltrating lymphocytes, and PD-L1 and PD-L2 expression on immune cells which can downregulate the patient’s immune response via PD-1 signaling [37]. Overexpression of PD-L1 and PD-L2 on tumor-infiltrating CD4 and CD8 T cells causes suppression of the host immune response [37]. Therefore, blocking the interaction between checkpoint ligands and their receptors can create a powerful oncologic response driven by the patient’s own immune system.

Therapeutic agents target the PD-1/PD-L1 axis function by inhibiting the receptor-ligand interaction. This blockade reactivates T-cells, enhancing their capacity to recognize and eliminate cancer cells [38]. The first anti-PD-1 immune checkpoint inhibitor (ICI) was approved by the Food and Drug Administration (FDA) in 2014 for the treatment of advanced melanoma [39]. Subsequent clinical trials evaluating the efficacy of PD-L1, PD-L2, and PD-1 inhibitors demonstrated success in the treatment of solid tumors such as advanced melanoma, non-small cell lung cancer, renal cell carcinoma, colorectal cancer, and endometrial cancer [40]. This concept has been demonstrated in pivotal Phase 2/3 trials that have altered treatment for advanced and recurrent EC.

### Single-agent immunotherapy for recurrent disease: phase 2 studies

#### KEYNOTE-158: pembrolizumab

Pembrolizumab initially demonstrated durable antitumor activity in the phase 2 basket study, KEYNOTE-158 (NCT02628067) [41,42]. This study included patients with metastatic and/or recurrent EC who had failed one prior line of therapy. The patients received pembrolizumab 200 mg intravenously every three weeks for up to 35 cycles with a total of 79 dMMR EC patients included. In group, 48% of patients had an objective response: 11 patients (14%) with a complete response, and 27 (34%) with a partial response. The median PFS was 13.1 months (95% CI 4.3–34.4) months, and the median OS was not reached. Pembrolizumab proved to be safe, with only 12 patients (11%) experiencing a grade 3 or 4 adverse event. This led to the accelerated FDA approval of pembrolizumab monotherapy for recurrent dMMR EC that had failed one prior line of therapy on 21 March 2022 [43].

#### GARNET trial: dostarlimab

The GARNET trial (NCT02715284) was a phase 1 trial examining the antitumor activity and safety profile of dostarlimab, a PD-1 inhibitor, in advanced solid tumors [44]. The cohort of patients with EC was split into two groups: patients with dMMR tumors and those with pMMR disease. Patients were given dostarlimab 500 mg every 3 weeks for 4 cycles, followed by dostarlimab 1000 mg every 6 weeks until progression. Patients in the dMMR cohort had an ORR of 43.5% (95% CI 34–53.4). In the mismatch repair proficient (pMMR) cohort, the ORR was 14.1% (95% CI: 9.1–20.6). The outcomes of the GARNET trial established the foundation for the RUBY trial and led to accelerated approval of single-agent dostarlimab for dMMR EC in April 2021.

*Summary*: Single-agent dostarlimab and pembrolizumab showed promising activity in dMMR EC leading to accelerated FDA approval and further phase 3 trials investigating combination immunotherapy and chemotherapy.

### Combining multiple immunotherapeutic agents

T-cell immunoglobulin and ITIM (TIGIT) domain is an inhibitory receptor expressed on lymphocytes and has recently gained attention as a target for gynecologic cancer immunotherapy. TIGIT downregulates the activity of both T and natural killer (NK) cells, playing a critical role in suppressing various stages of the cancer immunity cycle. Preclinical studies suggest that blocking TIGIT could reduce the progression of solid tumors through dual inhibition mechanisms [45].

#### KEYVIBE 005: vibostolimab and pembrolizumab in the recurrent setting

Vibostolimab, a TIGIT inhibitor, has been investigated as a potential combination therapy with pembrolizumab to treat patients with advanced dMMR EC who have not previously received anti-PD-L1 therapy. The phase 2 trial KEYVIBE-005 (NCT05007106) evaluated the efficacy of this dual blockade [46]. Patients with dMMR tumors received vibostolimab 200 mg and pembrolizumab 200 mg every three weeks for up to 35 cycles. The trial demonstrated a 65% ORR, with five patients (13%) achieving a complete response and 21 patients (53%) showing a partial response. Median PFS was 15 months (95% CI 8.1–15.6), while median OS was not reached (95% CI 16.1–NR). Since anti-PD-L1 therapies are now commonly used as first-line treatment, the pool of patients eligible for this combination in the recurrent setting is limited as there are no recommendations for the use of ICI after previous ICI therapy.

*Summary*: More research is needed to better understand combination immunotherapy for advanced and recurrent endometrial cancer.

### Immunotherapy in combination with chemotherapy for advanced and recurrent disease

#### NRG GY-018: pembrolizumab

Building on results from KEYNOTE-158, NRG GY-018 (NCT03914612) was a phase 3 trial that examined the addition of pembrolizumab to standard-of-care paclitaxel and carboplatin chemotherapy in patients with advanced or recurrent EC [47]. The arms contained two cohorts of patients that enrolled separately: a dMMR group and a pMMR group. The patients in the investigational arm received pembrolizumab 200 mg, paclitaxel 175 mg/m^2^ body surface area, and carboplatin AUC 5 mg every 3 weeks for 6 cycles followed by pembrolizumab 400 mg every 6 weeks for up to 24 months. Results of the dMMR group demonstrated a 70% reduction in the risk of disease progression or death in patients who received pembrolizumab compared to placebo (HR = 0.30; 95% CI: 0.41–0.71; *p* < 0.001). The median PFS in the dMMR group was not reached vs. 7.6 months in the placebo arm. The OS data presented at the Society of Gynecologic Oncology (SGO) conference in 2024 found no significant difference in OS (HR, 0.55; 95% CI, 0.25–1.19; *p* = 0.0617). Of note, 54.5% of patients in the placebo dMMR arm crossed over to the treatment arm after progression [48].

In the pMMR subgroup, there was a 46% reduction in the risk of progression or death compared to chemotherapy (HR = 0.54, 95% CI: 0.41–0.71; *p* < 0.001), with a PFS of 13.1 vs. 8.7 months. The median OS was 27.96 months in the pembrolizumab arm vs. 27.37 months in the placebo arm (HR, 0.79; 95% CI, 0.53–1.17; *p* = 0.1157). A significant proportion of trial crossover was observed, with 45% of patients in the pMMR placebo arm receiving immunotherapy [48]. In an exploratory subset analysis of the pMMR group, patients with a PD-L1 combined positive score (CPS) of 1 or higher had a median PFS of 13.1 months in the pembrolizumab arm, compared to 8.5 months in the placebo arm (HR = 0.59; 95% CI, 0.43–0.80). For patients with a PD-L1 CPS of less than 1, the median PFS was 15.1 months with pembrolizumab vs. 11.0 months with placebo (HR = 0.44; 95% CI, 0.26–0.75), concluding that the addition of pembrolizumab improved PFS regardless of PD-L1 status [47,48].

Based on these results, pembrolizumab is National Comprehensive Cancer Network (NCCN) recommended for all patients with advanced or recurrent EC irrespective of MMR status [49]. On 17 June 2024, the FDA approved pembrolizumab with carboplatin and paclitaxel followed by single-agent pembrolizumab for patients with advanced or recurrent EC regardless of MMR/MSI status [50].

#### RUBY part 1: dostarlimab

Part 1 of the RUBY trial examined the efficacy of dostarlimab in patients with primary advanced or recurrent EC in comparison with standard-of-care chemotherapy (NCT03981796). This phase 3 study administered dostarlimab 500 mg or placebo plus paclitaxel and carboplatin every 3 weeks for 6 cycles, followed by dostarlimab 1000 mg or placebo every 6 weeks for up to 3 years [51]. In comparison to GY-018, the dMMR group and overall cohort were the analytic groups and patients with carcinosarcomas were eligible. In patients with dMMR tumors, dostarlimab was associated with a 72% reduction in the risk of progression or death compared to chemotherapy alone (HR, 0.28; 95% CI 0.16–0.5; *p* < 0.001) and the probability of PFS at 24 months was 61.4% in the experimental group compared to 15.7% in the placebo group. The experimental arm also showed a PFS benefit in the overall population with a 36% reduction in the risk of progression or death (HR 0.64, 95% CI 13–23.9; *p* < 0.001). In an updated publication on overall survival, the use of dostarlimab in the overall population demonstrated an OS benefit of 44.6 months compared to 28.2 months in the chemotherapy-only group (HR 0.69, 95% CI 0.54–0.89; *p* = 0.002). The dMMR cohort receiving dostarlimab and chemotherapy had a median OS that was not reached, in contrast to 31.4 months for the chemotherapy group (HR: 0.32, 95% CI 0.17–0.63; *p* = 0.002) [52]. Initially, in July 2023, dostarlimab was FDA-approved for advanced and recurrent endometrial cancer that is dMMR; however, on 01 August 2024, the FDA approved the use of dostarlimab in an all-comer population [53,54].

#### ATTEND: atezolizumab

The ENGOT-en7/AtTEnd trial (NCT03603184) is a phase 3 randomized controlled trial that assessed the impact of adding atezolizumab, an anti-PD-L1 agent, to standard platinum-based chemotherapy. The experimental group received atezolizumab 1200 mg, paclitaxel, and carboplatin every three weeks for 6 to 8 cycles, followed by atezolizumab 1200 mg every three weeks until disease progression or toxicity [55].

Participants were divided into two analytical cohorts: dMMR and the overall population. In the dMMR group, median PFS was not reached with the addition of atezolizumab compared to 6.9 months in the chemotherapy-only arm. There was a 64% reduction in the risk of progression or death in the dMMR population (HR = 0.36; 95% CI: 0.23–0.57; *p* = 0.0005) and a 59% reduction in the risk of death (HR = 0.41; 95% CI: 0.22–0.76). In the overall population group, there was a 26% reduction in the risk of progression (HR = 0.74; 95% CI: 0.61–0.91; *p* = 0.0219), with a median PFS of 10.1 months vs. 8.9 months for chemotherapy alone. An interim OS analysis was performed among the all-comer population with only 43% data maturity and did not reach statistical significance (HR = 0.82; 95% CI: 0.63–1.07; *p* = 0.024). No differences in PFS or OS were observed between the atezolizumab and chemotherapy-alone groups for pMMR patients. Currently, atezolizumab does not have an FDA approval or NCCN recommendation for use in this setting.

*Summary*: Immunotherapy with chemotherapy should be considered the standard of care for all patients with dMMR mutations. While there is a benefit to patients with pMMR tumors and should be offered, the benefit is not as robust, and more research is needed to best understand the optimal treatment strategy for that group of patients.

### Immunotherapy in combination with chemoradiation in early high-risk and locally advanced endometrial cancer: KEYNOTE-B21

**Figure 1:**
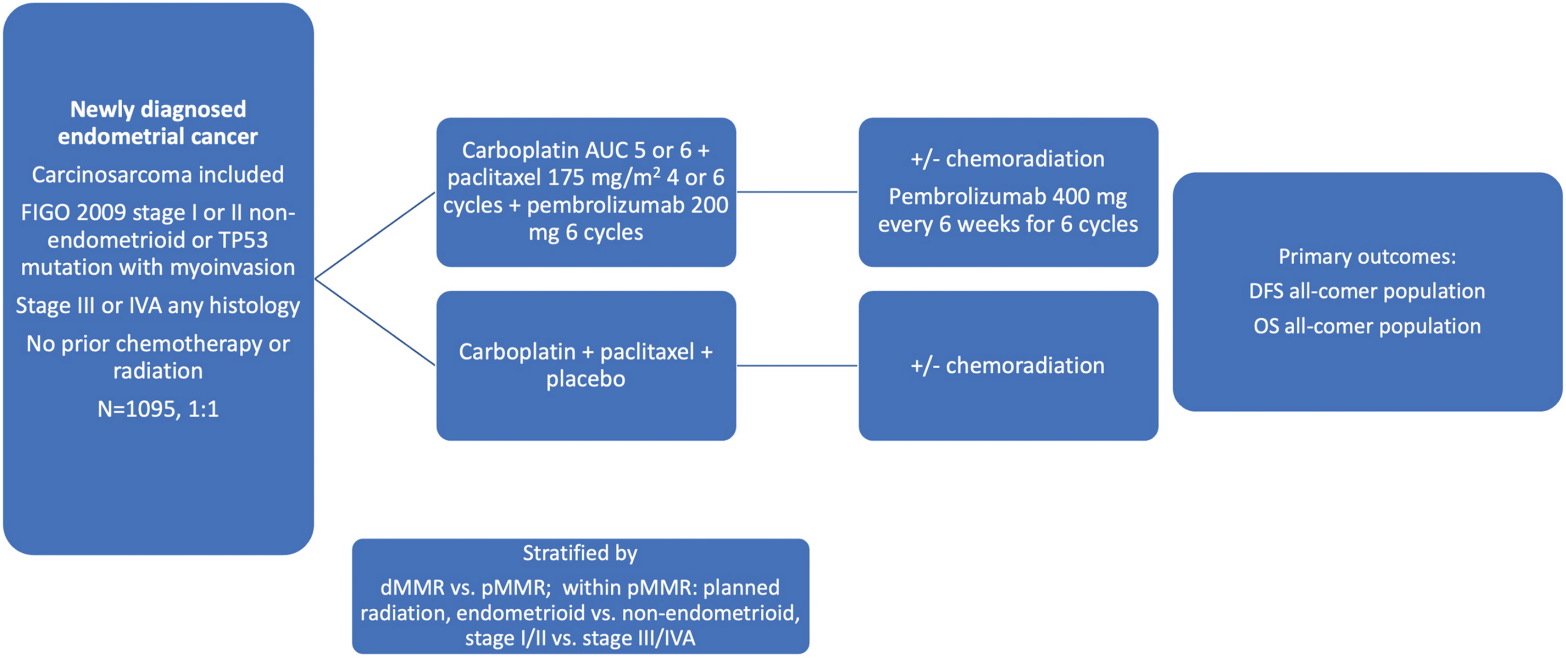
Schema for the KEYNOTE B-21 trial [56]. Abbreviations: DFS: disease-free survival, OS: overall survival, AUC: area under the curve

The incorporation of immunotherapy into earlier-stage high-risk and locally advanced endometrial cancer was recently investigated in the KEYNOTE-B21 phase 3 randomized control trial (NCT04634877) [56]. This trial enrolled a population similar to that of PORTEC-3 and GOG-258: non-endometrioid or TP53 mutant stage I and II patients with myometrial invasion, and stage III to IVA endometrial cancer of any histology (Fig. 1). Carcinosarcoma was included. An all-comer population was enrolled and stratified by MMR status. Patients were treated with chemotherapy consisting of carboplatin and paclitaxel for 4 to 6 cycles with the option of additional CRT or RT. The treatment arm was also given pembrolizumab 200 mg every 3 weeks for 6 cycles followed by pembrolizumab maintenance. The outcomes were disease-free survival (DFS) and overall survival. Over 1000 patients were randomized and no difference in DFS was seen at 24 months between groups in the overall intent-to-treat population (HR = 1.02, 95% CI: 0.79–1.32, *p* = 0.57). When looking at the dMMR subgroup, there was a signal toward improved DFS, although it was not an analytical endpoint in this study. At 24 months, 92% of dMMR patients treated with immunotherapy were disease-free, compared to 80% treated with placebo (HR: 0.31, 95% CI: 0.14–0.69) [57].

*Summary*: While it is hard to draw statistically meaningful conclusions from this study, it appears that there is a strong signal for the use of immunotherapy in the dMMR population in early-stage high-risk and locally advanced endometrial cancer, and trials are needed to further explore this. Given that this trial was not powered to determine a difference between the pMMR and dMMR subgroups, a separate trial powered to investigate early high-risk and locally advanced dMMR EC is needed to consider adjuvant immunotherapy as the standard of care in this population.

### Immunotherapy in combination with chemotherapy and PARP inhibition in advanced and recurrent endometrial cancer

Poly ADP-ribose polymerase (PARP) inhibitors have revolutionized the treatment of ovarian cancer, particularly for patients with Breast Cancer genes (BRCA 1/2) mutations or homologous recombination deficiency (HRD). PARP proteins play a role in repairing single-stranded DNA breaks and PARP inhibitors prevent these repairs leading to generation of double-stranded DNA breaks which cannot be repaired by cells with BRCA 1/2 mutations or HRD, leading to cell death [58]. Endometrioid grade 3 and high-risk histologic subtypes of EC are known to have increased rates of TP53, HER2, and loss of heterozygosity mutations. Specifically, TP53 mutated endometrial tumors are known to spread transperitoneally, similar to ovarian cancer [59]. The rationale for combining PARP inhibitors and ICIs stems from data demonstrating that PARP inhibitors upregulate PD-L1 expression in breast cancer cell lines and animal models [60]. It was thought that the co-use of these two drug classes could potentiate their efficacy while decreasing rates of resistance [61].

#### DUO-E trial: durvalumab and olaparib

The DUO-E trial (NCT04269200) randomized patients with newly diagnosed advanced or recurrent EC in a 1:1:1 ratio with the following arms: carboplatin and paclitaxel every 3 weeks for 6 cycles followed by placebo maintenance, carboplatin, paclitaxel plus durvalumab 1120 mg every 3 weeks for six cycles, followed by durvalumab maintenance of 1500 mg every 4 weeks, and lastly, carboplatin, paclitaxel plus durvalumab followed by durvalumab plus olaparib maintenance 300 mg tablets twice daily [62]. Patients were stratified based on their MMR status, and primary endpoints were PFS and OS for the durvalumab arm vs. control and the durvalumab plus olaparib arm vs. control. It is important to note that the trial was not designed to detect a statistical difference between the durvalumab and durvalumab and olaparib arms.

In the all-comer population, the durvalumab group had a 29% reduction in the risk of disease progression or death compared to the control group (HR 0.71, 95% CI 0.57–0.89; *p* = 0.003). The median PFS was 10.2 months and 9.6 months for the two groups, respectively. The durvalumab and olaparib group had a 45% reduction in the risk of disease progression or death compared to the control group (HR 0.55, 95% CI 0.43–0.69; *p* < 0.0001; median PFS 15.1 vs. 9.6 months). In an exploratory subgroup analysis, there was an improvement in PFS with the addition of olaparib to durvalumab in the pMMR population. There did not appear to be an added benefit with the addition of olaparib to durvalumab in the dMMR group, however, the study was not powered to address this question. In patients who were homologous recombination repair mutant (HRRm), the combination of durvalumab plus olaparib resulted in a 70% reduction in risk of disease progression (HR 0.3, 95% CI 0.15–0.58).

Overall survival data were shared at the SGO 2024 conference. Median OS had not been reached in the study groups. In the overall population, the addition of olaparib to durvalumab demonstrated a reduction in risk of death by 41% (HR 0.59, 5% CI 0.42–0.83; *p* = 0.003) while the durvalumab and the chemotherapy-only group had a 23% reduction (HR 0.77, 95% CI 0.56–1.07; *p* = 0.12). In the dMMR subgroup, there was a significant reduction in the risk of death by 66% (HR 0.34, 95% CI 0.13–0.79) in the durvalumab group and a reduction of 72% (HR 0.28, 95% CI 0.10–0.28) in the durvalumab plus olaparib arm. There was no statistically significant improvement in OS in the pMMR population [63]. Currently, durvalumab and chemotherapy is approved in the United States for the dMMR population only [64].

#### RUBY part 2: dostarlimab plus niraparib

Investigators of the Ruby Part 2 trial (NCT03981796) hypothesized that adding a PARP inhibitor in the maintenance phase may further improve outcomes in patients with pMMR endometrial cancer [65]. Patients with primary advanced or recurrent EC were randomized into two arms: carboplatin, paclitaxel, and dostarlimab 500 mg every 3 weeks for 6 cycles followed by dostarlimab 1000 mg every 6 weeks and niraparib 200–300 mg daily for up to 3 years compared to chemotherapy alone. Authors demonstrated a significant PFS advantage in the overall population with a 40% decreased risk of disease progression (HR = 0.60, 95% CI 0.43–0.82; *p* = 0.0007; PFS 14.5 vs. 8.3 months) in the experimental arm. Similar findings for PFS were found in the pMMR population, with an HR of 0.63 (95% CI 0.44–0.91; *p* = 0.006; PFS 14.3 months vs. 8.3 months).

Currently, there is no FDA approval in the United States for the use of PARP inhibitor therapy in the treatment of EC. Neither trial was statistically powered to demonstrate an incremental benefit with the addition of PARPi to immunotherapy so conclusions cannot be drawn regarding its superiority to immunotherapy alone. It appears that the addition of PARPi to immunotherapy may have some synergistic effect on the pMMR population, but more research needs to be done to understand who within that heterogeneous population derives the most benefit. The improved HR in the HRRm subgroup analysis in DUO-E is thought-provoking and warrants further investigation.

*Summary*: Combination immunotherapy, PARP inhibition, and chemotherapy shows treatment effects for patients with advanced and recurrent EC but more work is needed to understand the effect of each novel therapeutic and optimal patient selection while limiting side effects and cost.

The above trials have many similarities but also differ in their inclusion and exclusion criteria, statistical analysis, and endpoints. GY-018 excluded patients with carcinosarcoma, while the other trials included this population. Advanced disease was defined differently; GY-018 and DUO-E required patients with stage 3 disease to have measurable disease, while Ruby Part 1 only required measurable disease for stage 3 endometrioid histology. GY-018 and DUO-E required patients to have a disease-free interval of 12 months or greater while the other three trials only required a 6-month disease-free interval. GY-018 was statistically designed to show a difference between the dMMR and pMMR groups separately, while Ruby Part 1 and AtTEnd were designed to examine differences in the overall population and the dMMR subgroup. DUO-E was not designed to show a difference between the two experimental arms. These trials are summarized and results are compared in Table 3.

**Table 3:** Summary and comparisons of outcomes of recent phase 3 clinical trials in the treatment of advanced and recurrent endometrial cancer

Trial, Treatment		RUBY Part 1 [51], Dostarlimab (n = 494)	GY-018 [47], Pembrolizumab (n = 816)	AtTEnd [55], Atezolizumab (n = 549)	DUO-E [62], Durvalumab + olaparib (n = 718)		RUBY Part 2 [65], Dostarlimab + niraparib (n = 291)
Duration of use		3 years maintenance	2 years maintenance	Maintenance until progression	Maintenance until progression		3 years maintenance
Stage		Stage III (19%)	Stage III, IV or recurrent disease (% not reported)	Stage III (5.6%)	Stage III (6%)		Stage III (16%)
		Stage IV (34%)		Stage IV (27%)	Stage IV (41%)		Stage IV (33%)
		Recurrent (48%)		Recurrent (67%)	Recurrent (52%)		Recurrent (51%)
Histology		Endometrioid: 55%	Endometrioid: 59%	Endometrioid: 64%	Endometrioid: 60%		Endometrioid: 63%
		Serous: 21%	Serous: 19%	Serous: 16%	Serous: 21%		Serous: 15%
		Clear cell: 3%	Clear cell: 5%	Clear cell: 3%	Clear cell: 3%		Clear cell: 4%
		Carcinosarcoma: 8.9%		Carcinosarco ma: 9%	Carcinosarcoma: 7%		Carcinosarcom a: 9%
TFI		≥6 months	≥12 months	≥6 months	≥12 months		≥6 months
					**Durvalumab**	**Durva + olaparib**	
All comers	ΔPFS (months)	3.9 (7.9 vs. 11.8)	10.3 (8.5 vs. 18.8)	1.2 (8.9 vs. 10.1)	0.6 (9.6 vs. 10.2)	5.5 (9.6 vs. 15.1)	6.2 (8.3 vs. 14.5)
	HR (95% CI)	0.64 (0.51–0.80)	Not reported	0.74 (0.61–0.91)	0.71 (0.57–0.89)	0.55 (0.43–0.69)	0.60 (0.43–0.82)
dMMR	ΔPFS (months)	22.6 (7.7 vs. 30.3)	NR (7.6 vs. NR)	NR (6.9 vs. NR)	NR (7.0 vs. NR)	24.8 (7.0 vs. 31.8)	NR (7.9 vs. NR)
	HR (95% CI)	0.29 (0.17–0.50)	0.30 (0.19–0.48)	0.36 (0.23–0.57)	0.42 (0.22–0.80)	0.41 (0.21–0.75)	0.48 (0.24–0.96)
pMMR	ΔPFS (months)	2.0 (7.9 vs. 9.9)	4.4 (8.7 vs. 13.1)	0.3 (9.2 vs. 9.5)	0.2 (9.7 vs. 9.9)	5.3 (9.7 vs. 15.0)	6.0 (8.3 vs. 14.3)
	HR (95% CI)	0.76 (0.59–0.98)	0.54 (0.41–0.71)	0.92 (0.73–1.16)	0.77 (0.60–0.97)	0.57 (0.44–0.73)	0.63 (0.44–0.91)

Note: Abbreviations: PFS: progression-free survival, HR: hazard ratio, dMMR: mismatch repair deficient, pMMR: mismatch repair proficient, TFI: treatment free interval. Modified for use from Chan et al. Gyn Onc 2024 with permission [69].

Further, primary and secondary resistance to immunotherapy is common. Primary resistance is when patient tumors do not respond to upfront immunotherapy treatment. Rates of primary resistance are higher in tumors with low tumor mutational burden such as pMMR tumors [66]. Secondary resistance is common and occurs in about 25%–33% of patients who initially responded [47,51]. The underlying causes of secondary resistance can be complex and are multifactorial. These include changes in immune response and signaling pathways leading to an immunosuppressive environment [67]. Further, cancer cells can start to upregulate alternative inhibitory pathways or over-express PD-L1 to overcome inhibition [68]. Understanding mechanisms and predictors of immunotherapy resistance remains an active research need.

### Use of combination immunotherapy with targeted agents: tyrosine kinase and kinase inhibitors

Endometrial cancers that progress or recur following platinum-based chemotherapy are a treatment challenge, as there are no well-established guidelines for management. Non-platinum-based chemotherapy is often attempted, with generally poor effect. The introduction of targeted therapies has broadened treatment options for patients with recurrent EC. Lenvatinib, a multitargeted tyrosine kinase inhibitor, has shown some efficacy as a second-line treatment in this setting. In a single-arm, phase 2 trial by Vergote, lenvatinib was evaluated as a second-line therapy for patients with unresectable EC. The study reported an ORR of 14.3% (95% CI 8.8–21.4), with a median PFS of 5.6 months (95% CI 3.7–6.3) and a median OS of 10.6 months (95% CI 8.9–14.9) [70]. This led researchers to investigate its use in combination with other anti-cancer therapies.

#### KEYNOTE-775: lenvatinib and pembrolizumab in the recurrent setting

The combination of ICIs with lenvatinib has shown promising results in preclinical studies, outperforming either agent alone [71]. KEYNOTE-775 (NCT03517449) was a phase 3 randomized trial that evaluated combining lenvatinib with pembrolizumab in patients with advanced EC who progressed after at least one platinum-based chemotherapy regimen [72]. In the experimental arm, patients were administered lenvatinib 20 mg orally daily with pembrolizumab 200 mg intravenously every three weeks. This regimen was compared to the physician’s choice of chemotherapy, either doxorubicin 60 mg/m^2^ every three weeks or paclitaxel 80 mg/m^2^ weekly, with cycles of three weeks on and one week off. The primary endpoints of the study were PFS and OS in both the pMMR group and the overall population. In the pMMR group, the experimental arm demonstrated an improved PFS, with a median of 6.6 months (95% CI 5.6–7.4) compared to 3.8 months (95% CI 3.6–5.0) in the chemotherapy arm (HR = 0.60, 95% CI 0.5–0.72; *p* < 0.001). Lenvatinib and pembrolizumab improved OS in the overall and pMMR population (HR = 0.68, 95% CI 0.56–0.84; *p* < 0.001) leading to its FDA approval in July 2021 for patients with recurrent pMMR EC [73].

#### ENGOT-en9/LEAP-001: lenvatinib and pembrolizumab in the upfront setting

LEAP-001 (NCT03884101) is a phase 3 randomized trial that evaluated the efficacy of lenvatinib combined with pembrolizumab vs. standard paclitaxel and carboplatin as a first-line treatment for patients with newly diagnosed or recurrent advanced EC. Dual primary endpoints were PFS and OS for the pMMR and all-comer population with stratification based on MMR status. The study did not reach its primary endpoint for PFS or OS in either of the planned study populations. However, when looking at the exploratory dMMR population, there was an improvement in PFS (mPFS: 31.8 vs. 9.0 months, HR: 0.61, 95% CI: 0.40–0.92) with the use of lenvatinib and pembrolizumab compared to chemotherapy. A similar improvement in OS was noted (HR = 0.57, 95% CI: 0.36–0.91) [74]. These findings underscore the continued importance of chemotherapy in the initial treatment of EC. However, it generates the hypothesis that patients with dMMR tumors could potentially forgo cytotoxic chemotherapy in favor of combination targeted therapy, with more studies needed. Neoadjuvant pembrolizumab is an area of active investigation for dMMR tumors [75].

#### Futibatinib and pembrolizumab in the recurrent setting

As previously mentioned, pMMR tumors pose a significant treatment challenge due to the reduced efficacy of immunotherapy in this population. To address this, a phase 2 study (NCT05036681) is investigating the combination of futibatinib, a kinase inhibitor, and pembrolizumab in patients with metastatic pMMR EC. This combination is based on the observation that fibroblast growth factor receptor (FGFR) signaling is downregulated in many cancers. Preclinical data have shown that futibatinib selectively inhibits the growth of several tumor types, including EC [76]. The study is currently accruing participants.

*Summary*: Lenvatinib and pembrolizumab should be a treatment option for patients with pMMR tumors in the recurrent setting after progression with standard platinum-based chemotherapy. However, the use of pembrolizumab in the frontline setting limits use. More research is needed for immunotherapy and tyrosine kinase inhibitor use in the front-line setting, especially in the dMMR population.

### Other novel biomarker-based treatments in advanced and recurrent endometrial cancer

#### HER-2

Biomarker-driven therapy has gained traction in the last several years as new data has emerged showing targeted therapy can improve clinical outcomes and prognosis [77,78]. One relevant biomarker in EC is human epidermal growth factor receptor 2 (HER2/neu), a cell membrane receptor coded by ERBB2. HER2 is frequently overexpressed in a wide range of malignancies including EC and is associated with more aggressive disease and worse survival [79,80]. HER2 amplification is frequently found in clear cell, serous, and carcinosarcoma histologies with incidence ranging from 16%–80% in the literature [80–83].

Several recent clinical trials have investigated targeting HER2. In 2018, Fader et al., conducted a randomized phase 2 trial assessing the efficacy of trastuzumab, a monoclonal antibody to HER2/neu, in primary advanced or recurrent serous EC with overexpression of HER2/neu (NCT01367002) [84,85]. The study enrolled 61 patients and randomized them to platinum chemotherapy (carboplatin AUC 5, paclitaxel 175 mg/m^2^) vs. chemotherapy and trastuzumab 8 mg/kg for the first dose followed by 6 mg/kg in subsequent cycles until progression or toxicity with PFS as the primary outcome. Median PFS was improved by 4.6 months in the experimental vs. control arm (12.6 vs. 8.0 months, *p* = 0.005; HR 0.44; 90% CI 0.26–0.76). The benefit of trastuzumab was found in both the primary (*p* = 0.013; HR 0.40; 90% CI 0.20–0.80) and recurrent (*p* = 0.003; HR, 0.14; 90% CI, 0.05–0.54) settings. Overall survival was improved with trastuzumab with a median OS of 29.6 months vs. 24.4 months (HR 0.58, 90% CI 0.34–0.99, *p* = 0.046) with the greatest benefit in patients with primary advanced disease.

More recently, the DESTINY-PanTumor02 (NCT04482309) trial evaluated the safety and efficacy of Trastuzumab Deruxtecan (T-DXD), a HER2-directed antibody-drug conjugate (ADC) in patients with HER2-expressing solid tumors with locally advanced or metastatic disease [86]. Patients were required to have had at least one prior line of treatment. T-DXD (5.4 mg/kg) was given every 3 weeks until progression with 40 patients with EC enrolled. The ORR in the EC group was 57.5% (95% CI 40.9%–73.0%), the highest of all disease site cohorts. When stratified by IHC status, the response rate was most pronounced in patients with 3+ IHC staining (ORR 84.6%) vs. 2+ IHC staining (ORR 47.1%). Neither median PFS (95% CI: 7.3 months-NR) nor OS (95% CI: 18.9-NR) was reached in the 3+ group. In the overall population, both patients who had received prior HER2 therapy (n = 38; ORR: 36.8% [95% CI, 21.8 to 54.0]) and those who had not received HER2-directed therapy (n = 227; ORR: 37.4% [95% CI, 31.1 to 44.1]) benefitted. Post hoc analyses found no difference in central compared to local IHC testing and other researchers have demonstrated the feasibility of plasma cell-free DNA testing for HER-2 [87,88].

Another HER-2 targeting agent, trastuzumab duocarmazine uses the prodrug seco-duocarmycin-hydroxybenzamide-azaindole as the cytotoxic linker. Preclinical models have demonstrated it to be more cytotoxic for low HER-2 tumors [89]. A phase 1 dose expansion trial of patients with a solid tumors with at least HER-2 1+ expression was performed. In total 14 EC patients were enrolled with a partial response seen in 5 patients (39%, 95% CI: 13.9–68.4) [90]. This warrants further investigation, especially for the HER-2 low-expressing population. HER-2 targeting ADCs have demonstrated activity in HER-2 low-expressing breast cancers, an area of future research for EC [91].

Interstitial lung disease (ILD) or pneumonitis are potentially serious adverse events with T-DXD treatment, with an incidence of 10%–12% [91]. Patients should be screened with a chest CT scan before starting and then every 12 weeks while on treatment. Grade 2 and 3 adverse events require permanent discontinuation of T-DXD. Grade 1 adverse events should result in temporary discontinuation of T-DXD with administration of steroids, and if pneumonitis does not improve by day 49 of treatment or after day 22 of treatment, T-DXD should also be permanently discontinued [92].

**Figure 2:**
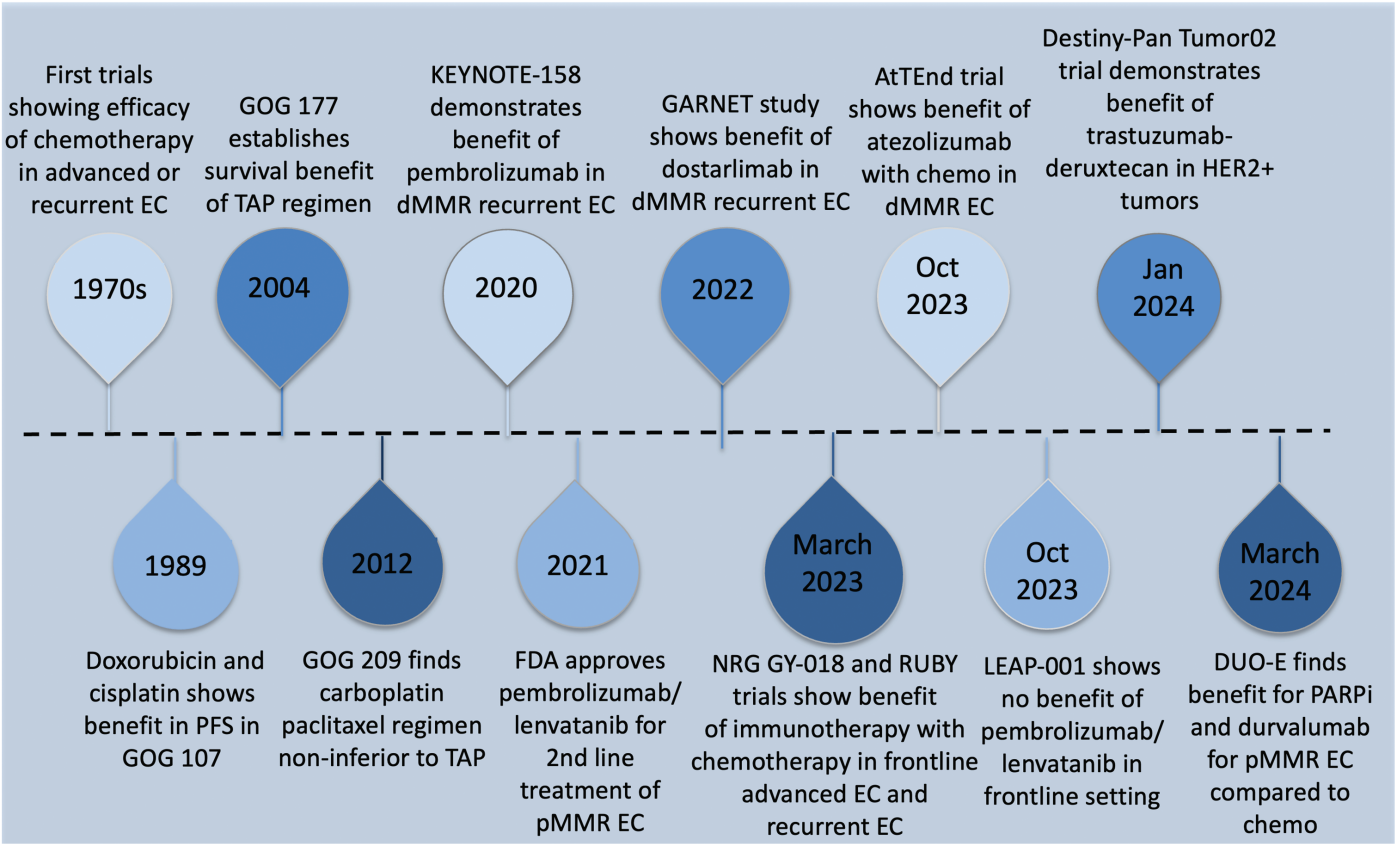
Timeline summary of key developments in the treatment of advanced and recurrent endometrial cancer

**Figure 3:**
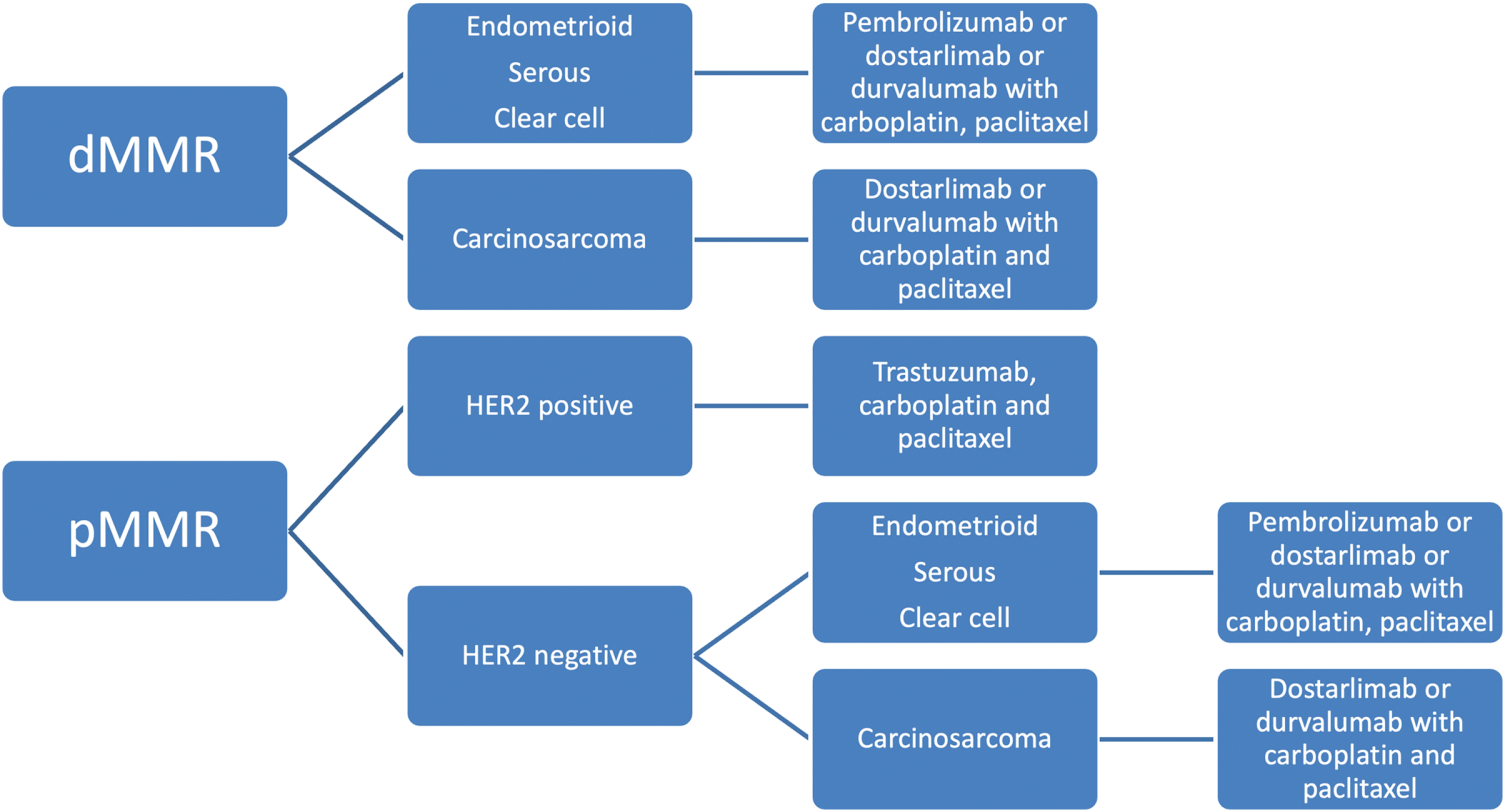
Treatment algorithm for upfront advanced endometrial cancer based on current FDA drug approvals

**Figure 4:**
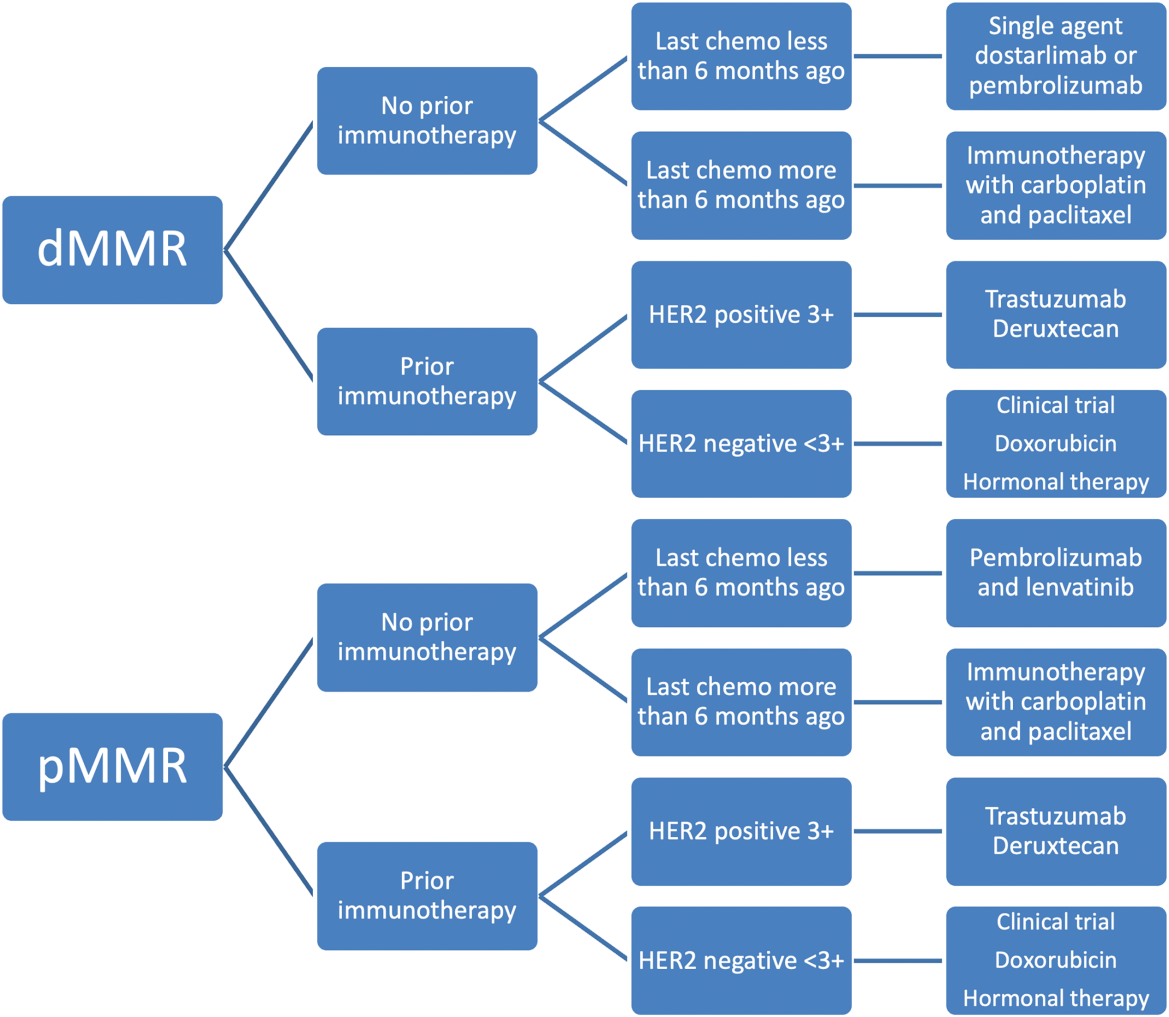
Treatment algorithm for recurrent endometrial cancer based on current FDA drug approvals

T-DXD received accelerated approval from the FDA in 2024 for all unresectable or metastatic HER2 overexpressing solid tumors with 3+ immunohistochemistry (IHC) expression [93]. Patients who are HER2 equivocal (2+) by IHC should undergo in situ hybridization testing to test for overexpression. All FDA-approved therapeutic agents for primary advanced or recurrent endometrial cancer are listed in Table 4. A summary of the history of therapeutic development in endometrial cancer is reviewed in Figs. 2–4 delineate treatment algorithms for primary advanced and recurrent EC respectively, based on current FDA-approved medications.

**Table 4:** Summary of FDA-approved medications and their indications in endometrial cancer

Drug name	Class of drug	Indication	Key trial
Carboplatin	Platinum-based chemotherapeutic agent	Used in combination with paclitaxel for treatment of advanced or recurrent endometrial cancer.	GOG-209 [15]
			NCT00063999
Paclitaxel	Taxane chemotherapeutic agent	Used in combination with carboplatin for the treatment of advanced or recurrent endometrial cancer.	GOG-209 [15]
			NCT00063999
Pembrolizumab	PD-1 inhibitor	Approved as monotherapy for recurrent dMMR endometrial cancer that has failed one prior line of therapy.	Keynote-158 [42]
			NCT02628067
Pembrolizumab	PD-1 inhibitor	Approved in combination with carboplatin/paclitaxel followed by pembrolizumab monotherapy for advanced or recurrent endometrial cancer regardless of MMR/MSI status.	NRG GY-018 [47]
			NCT03914612
Durvalumab	PD-L1 inhibitor	Approved in combination with chemotherapy for patients with dMMR primary advanced or recurrent endometrial cancer.	DUO-E [62]
			NCT04269200
Dostarlimab	PD-1 inhibitor	Approved as monotherapy for recurrent dMMR endometrial cancer that has failed one prior line of therapy.	GARNET [44]
Dostarlimab	PD-1 inhibitor	Approved for advanced or recurrent endometrial cancer regardless of MMR/MSI status.	RUBY Part 1 [51]
			NCT03981796
Lenvatinib	Tyrosine kinase inhibitor	Approved in combination with pembrolizumab in recurrent pMMR/MSI-S endometrial cancer.	KEYNOTE-775 [72]
			NCT03517449
Trastuzumab deruxtecan	HER-2 directed antibody-drug conjugate	Agnostic approval for patients with unresectable or metastatic HER2-positive (IHC 3+) solid tumors who have received prior systemic treatment and have no satisfactory alternative options.	DESTINY-PanTumor02 [86] NCT04482309


#### Uterine carcinosarcoma and HER-2

Uterine carcinosarcoma (UCS) is a rare type of EC that has a notoriously poor prognosis and few treatment options beyond standard platinum chemotherapy [94]. HER2 amplification has been found in 15%–32% of UCS, suggesting a therapeutic target [95,96]. The STATICE trial (NCCH1615), published in 2023, is a phase 2 single-arm trial that evaluated the efficacy and safety of T-DXD in HER-2-expressing advanced or recurrent UCS [97]. To qualify for inclusion, patients must have previously received standard chemotherapy and have at least 1+ HER2 expression on IHC staining. Subsequently, they were stratified into HER2-high (IHC 2+ and 3+) and HER2-low (IHC 1+) groups. Thirty-four patients were enrolled and received T-DXD 6.4 or 5.4 mg/kg (weight-based) once every 3 weeks until toxicity or progression. The primary endpoint was ORR in the HER2-high group. In the HER2-high group, the ORR was 54.45% (95% CI, 32.2–75.6), while the ORR in the HER2-low group was 70.0% (95% CI, 34.8–93.3). This study shows promise for the use of HER2-targeted therapies in UCS, even in patients with only 1+ expression on IHC.

*Summary*: HER-2 is a promising and targetable biomarker for EC. Patients with HER-2-positive tumors could be treated with trastuzumab in addition to platinum-based chemotherapy in the front-line setting. Patients with HER-2 3+ tumors should receive trastuzumab deruxtecan in the recurrent setting. Those with HER-2 2+ tumors on IHC should undergo *in situ* hybridization. More research is needed for optimal treatment sequencing and understanding the utility of treating HER-2 low and equivocal tumors.

#### TP53 wildtype

Another therapeutic target under investigation in advanced or recurrent EC is Exportin-1 (XPO1). XPO1 is a protein responsible for the export of hundreds of proteins including tumor suppressor proteins TP53, PTEN, and FOXO1 [98–100]. Selinexor is a selective inhibitor of XPO1 that has been FDA-approved for select hematologic malignancies but has recently shown promise in the maintenance phase of advanced or recurrent EC. The SIENDO trial (NCT03555422), was a randomized, phase 3 trial enrolling 263 patients with advanced or recurrent EC [101]. All patients received at least 12 weeks of standard chemotherapy. They were then randomized 2:1 to receive Selinexor (60 mg or 80 mg weekly) vs. placebo in 28-day cycles until disease progression or discontinuation with the primary outcome of PFS. On initial analysis, there was no statistically significant difference in PFS between selinexor and placebo (PFS 5.7 months vs. 3.8 months, HR 0.76, 95% CI 0.54–1.08; *p* = 0.126). However, a prespecified exploratory analysis for incorrect chemotherapy response data showed an improvement in median PFS with a 29% reduction in the risk of progression (HR 0.71, 95% CI: 0.499–0.966, *p* = 0.049). PFS was analyzed within TCGA groups and patients with TP53 WT tumors appeared to benefit the most (median PFS 13.7 vs. 3.7 months, HR: 0.38, 95% CI 0.21–0.67).

Long-term follow-up from the TP53 WT subgroup found an improved PFS in patients receiving selinexor maintenance; 28.4 months vs. 5.2 months in the placebo group (HR 0.44; 95% CI 0.27–0.73) [102]. The benefit was more pronounced in the pMMR cohort (39.5 vs. 4.9 months, HR 0.36; 95% CI 0.19–0.71) than in the dMMR cohort (13.1 vs. 3.7 months, HR 0.49; 95% CI 0.18–1.34). A confirmatory phase 3 trial of selinexor maintenance for TP53 WT patients, GOG-3083, is underway given the findings in this subgroup analysis [103] (NCT05611931) (Table 1). This could be a meaningful treatment option for patients with TP53 WT pMMR tumors as the benefit from immunotherapy is less pronounced compared to patients with dMMR tumors. While it is challenging to conclude from cross-trial comparisons, the mPFS in the pMMR selinexor group is much greater than the mPFS in the ICI and PARPi trials in the pMMR population (39.5 vs. 9.5–15.0 months). This warrants further exploration in the TP53 WT pMMR population. It is unclear how selinexor maintenance would affect the treatment of the TP53 WT dMMR population given the well-established benefit of ICI.

#### TROP-2

Trophoblast cell surface antigen 2 (TROP-2) is a protein that is commonly overexpressed in more aggressive endometrial cancers including grade 3 endometrioid and uterine serous tumors [104]. TROP-2 is a newer target currently under investigation for persistent or recurrent EC [104,105]. Initial data on the use of sacituzumab govitecan, an ADC directed at TROP-2 in patients with persistent or recurrent EC was recently published. The TROPiCS-03 (NCT03964727) was a phase 2 basket trial of metastatic solid tumors including EC [106]. In total 41 patients with recurrent EC with previous platinum chemotherapy and immunotherapy were treated with sacituzumab govitecan 10 mg/kg on day 1 and 8 every 21 days. An ORR of 22% (95% CI: 11%–38%) was reported. An additional 44% had stable disease with a median duration of response of 8.8 months. Preliminary results from a phase 2 trial of sacituzumab irinotecan in advanced EC and OC (NCT 04152499) presented in September 2024 demonstrated encouraging findings. This was a similarly heavily pretreated cohort with 35% of patients receiving prior immunotherapy. Among the 44 EC patients enrolled, the ORR was 34.1%, and the disease control rate was 75%. The median PFS was 5.7 months [107]. Currently, there are two open phase 3 trials investigating TROP-2 therapy in EC (NCT06132958, NCT06486441) (Table 1). These trials require prior platinum chemotherapy and immunotherapy.

*Summary*: XPO1 inhibitors and TROP2 ADCs represent promising fields of research in the field of advanced and recurrent EC with more data from phase 3 studies needed.

## Role of mTOR, PIK3CA Pathways, and Hormonal Blockade in Advanced and Recurrent Endometrial Cancer

Estrogen is another area of interest in the landscape of targeted therapy in EC. Endometrioid endometrial carcinomas frequently exhibit estrogen (ER) and progestin receptor (PR) positivity [108–110]. Estrogen in EC activates the phosphoinositide 3-kinase (PI3K)/AK strain transforming (AKT)/mammalian target of the rapamycin (mTOR) pathway via upregulation of insulin-like growth factor 1 (IGF-1) [111–113]. Estrogen amplification also activates the RAS/RAF/MEK pathway, leading to cell growth and proliferation [113,114]. Additionally, EC tumors with ER/PR positivity frequently demonstrate mutations in PTEN, a tumor suppressor gene that independently suppresses activation of the PI3K/AKT/mTOR pathway [16,115]. As a result of the upregulation of the these pathways, cell cycle proliferation is activated via cyclin-d and cyclin-d kinase 4/6 (CDK 4/6) [116,117]. These pathways contain multiple potential targets for drug development.

Patients with hormone receptor-positive breast cancer have shown benefits with combined hormonal treatment and CDK inhibition, leading to FDA approval of three CDK inhibitors, palbociclib, ribociclib, and abemaciclib in this population [117–119]. Several trials are now aiming to target these pathways in EC. The PALEO trial was a randomized, phase 2 trial that evaluated the safety and efficacy of palbociclib, an oral selective inhibitor of CDK 4/6, in combination with letrozole, an aromatase inhibitor, vs. letrozole alone in patients with ER-positive recurrent or advanced endometrioid EC (NCT02730429) [120]. Seventy-three patients were randomized 1:1 to letrozole (2.5 mg PO daily for d1-28) with palbociclib (125 mg PO d1-21) vs. letrozole at the same dose with placebo (PO d1-21) until progression with a primary endpoint of PFS. Letrozole and palbociclib significantly improved PFS (8.3 vs. 3.1 months) in comparison to letrozole and placebo (HR 0.57, CI 0.32–0.99, *p* = 0.044). However, over two-thirds of patients on palbociclib required temporary interruption in treatment, and almost half required dose reduction due to adverse effects, most commonly neutropenia. The results of this trial are promising but demonstrate a need for dose optimization and a phase 3 trial is planned.

Similarly, Konstantinopolous et al., evaluated the efficacy of the combination of hormonal therapy and CDK inhibition using abemaciclib [121]. In this phase 2, single-arm, two-stage study, 30 patients with advanced and recurrent ER-positive (>=1% on IHC) EC were given letrozole 2.5 mg and abemaciclib 150 mg BID combination treatment. The primary endpoint, ORR, was 30% (95% CI, 14.7–49.4) with all responses in endometrioid tumors and a PFS of 55.6% at 6 months (95% CI, 35.1–72).

Preliminary data from another phase 2, single-arm study assessing the safety and efficacy of fulvestrant, an ER antagonist, in combination with abemaciclib, a CDK 4/6 inhibitor, was presented at ASCO 2024 (NCT03643510) [122]. Patients included in the study had ER-positive disease (>=1% on IHC), ≤2 prior lines of chemotherapy, and ≤1 prior line of hormonal therapy. Patients received 500 mg fulvestrant monthly with a 2-week loading dose in combination with abemaciclib 150 mg twice daily until progression or toxicity with a primary endpoint of ORR. An ORR of 44% (90% CI, 27.0–62.1) was observed with a median duration of response of 15.6 months (90% CI, 7.2—nonestimable (NE)). Notably, all responses were found in patients with G1 and G2 endometrioid EC who were classified in the TCGA as NSMP. Given these encouraging findings, a phase 3 trial is planned.

The findings in the above trials demonstrate that there are subsets of heavily pretreated patients that respond well to targeting the ER/PR pathway, particularly the G1 and G2 endometrioid tumors. These tumors likely represent part of the pMMR subgroup and more work is needed to further explore the molecular profiles of responders to endocrine therapy and design randomized phase 3 trials in these populations.

*Summary*: Hormonal and endocrine-based therapy has a role in the advanced or recurrent setting of EC particularly in patients that are ER/PR positive. Consideration for endocrine therapy should be made when patients have progressed after standard front-line therapeutics. More phase 3 trials are needed.

### Radiation and its role in novel therapeutics

The use of radiation with immunotherapy could have synergistic effects. The abscopal effect was first described by RH Mole, where it was observed that radiation directed at one region of the body can cause regression of tumors outside of the field. Radiation can increase neoantigen expression and enhance immune system activity against cancer cells [123,124]. The recent results of KEYNOTE-B21 did not show an improvement in any of the subgroups who received radiation but was also an overall negative study. There are several ongoing phase 1 or 2 clinical trials looking at the combination of radiation and immunotherapy in the recurrent, or advanced unresectable setting (NCT03955978, NCT05603910) (Table 1).

Post-operative chemoradiation or chemotherapy alone is a consideration for patients with high-risk or locally advanced (FIGO stage III) endometrial cancer based on several randomized clinical trials. Chemoradiation (CRT) has similar activity to chemotherapy in high-risk or locally advanced endometrial cancer as demonstrated in PORTEC-3 and GOG-258 and may provide some additional local and nodal control over chemotherapy alone [26,28]. Updated survival analysis from GOG-258 demonstrated improved local control with adjuvant chemoradiation but no difference in overall survival in any group [125]. In general, early-stage high-risk or locally advanced endometrial cancer should receive either adjuvant chemotherapy or radiation. Those with dMMR tumors could be considered for adjuvant pembrolizumab but more randomized, prospective data is needed.

### Future Directions

Currently, there is no data to support the use of ICI rechallenge in patients with EC previously treated with ICI. However, evidence exists from studies on non-small cell lung cancer (NSCLC) and melanoma regarding the efficacy of ICI rechallenge [126,127]. Studies have shown that pembrolizumab retreatment improves overall survival in patients with recurrent NSCLC compared to docetaxel [128]. The key challenge is identifying the right patient population for immunotherapy retreatment. Just as platinum sensitivity serves as a predictor for chemotherapy, it raises the question of whether a similar predictive marker exists for immunotherapy.

The optimal duration of maintenance immunotherapy for EC remains uncertain, with most trials administering maintenance therapy for approximately two years. The decision to treat patients for a set duration in these studies is shaped by several factors, including tolerability, adverse events, efficacy data from previous trials, and prespecified endpoints such as PFS and OS. Insights from trials like CheckMate-153 (NCT02066636), which evaluated continuous vs. one-year fixed-duration ICI therapy in previously treated NSCLC, have provided some guidance. The study found that median PFS was longer in the continuous treatment arm compared to the fixed one-year duration [129]. Opinions vary among experts regarding the appropriate duration of immunotherapy, a topic that requires more research.

The timing of immunotherapy in combination with chemotherapy also needs more investigation. A prospective, open-label clinical trial investigating the use of neoadjuvant chemotherapy and immunotherapy in unresectable, advanced, metastatic EC is currently being conducted [130]. A preliminary phase 1 trial of neoadjuvant immunotherapy in dMMR endometrial cancer demonstrated a pathologic response in 50% of patients [75]. Impressive response rates in patients with locally advanced dMMR colorectal cancer have been reported, again demonstrating the need for further research [131].

Further incorporation of the molecular classification of EC in the treatment paradigm is an important area of active investigation. The RAINBO trial (NCT05255653) is a randomized phase 3 trial currently enrolling patients in Europe examining this question [132]. They are enrolling four separate cohorts based on a patient’s TCGA molecular classification. For patients with TP53 mutations, adjuvant CRT and olaparib will be compared to CRT alone. For the ER-positive or NSMP tumors, radiation followed by hormonal therapy will be compared to adjuvant CRT. For patients with dMMR, adjuvant radiation, and durvalumab will be compared to radiation. Finally, patients with POLE hypermutated tumors will be treated with observation for low-risk disease and observation compared to radiation in high-risk disease. Ongoing relevant trials in advanced and recurrent EC are summarized in Table 1.

More research is needed to further understand the best-targeted treatment options for patients. Chemotherapy and ICI do not work as well for patients with pMMR tumors compared to dMMR tumors. In future studies, pMMR tumors need to be considered in the context of other potentially targetable mutations such as TP53, HRD, POLE, ER/PR, and NSMP as they likely represent a heterogeneous group of tumors. Further refinement of these molecular subtypes may help better understand which pMMR tumors may benefit from immunotherapy. Selinexor may be an alternative treatment option for patients with pMMR TP53 wild-type tumors, given recent promising data. However, patients with pMMR TP53 mutant tumors represent an area of need for future research. A subset analysis of GOG-86P demonstrated that the addition of bevacizumab to standard chemotherapy may result in survival benefits in patients with TP53 mutations [133]. The combination of bevacizumab, immunotherapy, and chemotherapy may be a future research direction for this subset of patients with pMMR TP53 mutant advanced or recurrent EC. The role of PARPi needs to be further investigated in EC, especially in the context of homologous recombination-deficient patients.

Finally, the cost of novel therapeutics remains a large global issue and applies to many of the therapeutics discussed in this review. Patients in low-income countries may not have access to new anti-cancer therapies or may have to pay for them themselves [134]. The cost-effectiveness ratios of novel therapeutics remain high with the cost of treatment exceeding 200,000 US dollars per year [135,136].

### Strengths and Limitations

This review contains several strengths and limitations. This is a comprehensive review bridging the literature in treatment paradigms from locally advanced to widely metastatic and recurrent EC. It contains the most up-to-date phase 3 research published or presented in the field. Limitations of the review include the differences in trial design, enrollment, subsequent treatment, and follow-up that limit the ability to perform cross-trial comparisons. Some trial data are still maturing and could impact results. Finally, phases 1 and 2 trials included in this review were by expert clinical opinion and are not comprehensive of the early phase research in the field of EC.

## Conclusions

In conclusion, there have been a plethora of recent advances in the treatment landscape of advanced and recurrent endometrial cancer. Many, if not all of the advances are driven by a deeper understanding of the molecular and genetic underpinnings of endometrial cancer and creating therapies targeting these alterations. The results generated from these studies also demonstrate how much more investigation is needed.

## Data Availability

All data generated or analyzed during this study are included in this published article.
